# Transcutaneous tibial nerve stimulation for the treatment of bladder storage symptoms in people with multiple sclerosis: Protocol of a single-arm feasibility study

**DOI:** 10.12688/hrbopenres.13107.1

**Published:** 2020-09-16

**Authors:** Hawra B. Al Dandan, Rose Galvin, Katie Robinson, Dorren McClurg, Susan Coote

**Affiliations:** 1 School of Allied Health, Faculty of Education and Health Sciences, Clinical therapies, University of Limerick, Limerick, County Limerick, Ireland; 2 College of Applied Medical Sciences, Physiotherapy, Imam Abdulrahman Bin Faisal University, Dammam, Saudi Arabia; 3 Health Research Institute, University of Limerick, Limerick, County Limerick, Ireland; 4 Aging Research Centre, University of Limerick, Limerick, County Limerick, Ireland; 5 Nursing, Midwifery and Allied Health Professions Research Unit, Glasgow Caledonian University, Glasgow, UK

**Keywords:** Multiple sclerosis, Neurogenic bladder, urinary symptoms, feasibility study, Tibial nerve stimulation.

## Abstract

**Background:** Neurogenic lower urinary tract dysfunction (NLUTD) is common among people with multiple sclerosis (MS) with a pooled prevalence of 68.41% using self-report measures and 63.95% using urodynamic studies. Transcutaneous tibial nerve stimulation (TTNS) is a non-invasive option to manage bladder storage symptoms; however, the potential efficacy of TTNS among people with MS is based on a small number of studies with the absence of high-quality evidence relating to efficacy, and lack of clarity of the optimal electrical stimulation parameters and frequency, duration and number of treatment sessions. This study aims to assess whether TTNS is feasible and acceptable as a treatment for bladder storage symptoms in people with MS.

**Methods:** We will use a single-arm experimental study to explore the feasibility and acceptability of TTNS in the treatment of bladder storage symptoms in MS. The CONSORT extension for pilot and feasibility studies will be followed to standardise the conduct and reporting of the study. The recruitment plan is twofold: 1) Open recruitment for people with MS through MS Ireland’s communication channels; 2) recruitment from a convenience sample of people with MS who have previously participated in a qualitative interview study of urinary symptoms. We will assess recruitment/retention rates, the urinary symptoms changes and the effect on quality of life pre and post intervention using ICIQ-OAB, 3-day bladder diary, King’s Health Questionnaire and collect self-reported data on adherence and adverse events. Acceptability of using TTNS will be evaluated at the end of intervention. This study has been reviewed and approved by the Education and Health Science’s Faculty Research Ethics Committee, University of Limerick [2020_06_07_EHS].

**Conclusion: **It is anticipated that assessing the feasibility and acceptability of TTNS for storage bladder symptoms in MS will inform the development of a definitive randomised trial.

**Trial registration**: ClinicalTrials.gov NCT04528784 ****27/08/2020

## Background

Neurogenic lower urinary tract dysfunction is defined by the International Continence Society (ICS) as “lower urinary tract symptoms (LUTSs) in the presence of a relevant neurological disease” ( Gajewski *et al*., 2018). Storage symptoms associated with LUTS include frequency, urgency, nocturia, and/or incontinence; voiding symptoms resulting in hesitancy, slow stream, intermittency, dysuria, straining, terminal dripple, need to immediately re-void, splitting or spraying whereas post micturition symptoms include incomplete bladder emptying and/or post micturition dribble ( Gajewski *et al*., 2018). The range and severity of neurogenic bladder symptoms depend on the location of the neurologic lesions ( Li *et al*., 2016; Peter & Peter, 2012), with more pronounced symptoms in people with MS associated with lesions at the pontine micturition centre (PMC) leading to storage symptoms and or the supra sacral lesions resulting in both storage and voiding symptoms accompanied with an increased post-void residual ( Haylen *et al*., 2010; McCombe *et al*., 2009; Panicker *et al*., 2015). 

We recently undertook a systematic review and meta- analysis which demonstrated that LUTSs are prevalent in people with MS with a pooled prevalence of 68.41% using self-report measures and 63.95% using urodynamic studies. When considering types of LUTSs, urinary frequency was the predominant symptom followed by urgency with a pooled prevalence estimate of 73.45% and 63.87% respectively using self-report measures. Detrusor overactivity was found to be the most prevalent urodynamic symptom, with a pooled prevalence estimate of 42.9% followed by detrusor sphincter dyssynergia at 35.44% ( Al Dandan *et al*., 2020). The high prevalence of urinary symptoms among people with MS is concerning as several studies have highlighted the negative consequences of urinary symptoms for quality of life ( Browne *et al*., 2015; Finlayson *et al*., 2006; Nortvedt *et al*., 2001; Vitkova *et al*., 2014). Studies have shown that urinary incontinence is considered as one of the worst morbidities associated with MS ( Fowler *et al*., 2009; Nakipoglu *et al*., 2009). Urinary symptoms including loss of urine that results in fear of leaking in public is considered as a barrier to engaging in physical activities among people with MS ( Kayes *et al*., 2011). Furthermore, an increased risk of mortality and an aggravation of MS symptoms following an episode of urinary tract infection (UTIs) has been reported ( Abrams *et al*., 2017). Timely diagnosis and proper management are of utmost importance in maintaining good quality of life among people with MS and urinary symptoms.

Current therapeutic interventions for storage symptoms include pharmacologic therapy, behavioural interventions, pelvic floor muscle training (PFMT), and/ or neuromodulation. Pharmacologic interventions such as the use of anticholinergic medication are considered a first-line treatment for neurogenic bladder population ( Chapple *et al*., 2008; Tornic & Panicker, 2018). Anticholinergic short-term efficacy has been reported in previous studies, however, limited long-term efficacy due to side effects and lack of tolerance was reported ( de Seze *et al*., 2011; Fowler *et al*., 2009; Li *et al*., 2016; Panicker *et al*., 2015; Stöhrer *et al*., 2009; Tornic & Panicker, 2018; Valles-Antuna *et al*., 2017). The reported side effects include; gastrointestinal tract disorders, central nervous system particularly worsening in cognition and deterioration in memory, dry mouth, impact on complete bladder emptying and increase cost.

A systematic review identified very few high quality studies of non-pharmacological interventions such as behavioural interventions including management of fluid intake, bladder retraining and scheduled voiding among a neurogenic population including people with MS ( Panicker *et al*., 2015). Combined pelvic floor muscle training (PFMT) therapy with electromyography (EMG) biofeedback and intravaginal neuromuscular electrical stimulation (NMES) reduced urinary symptoms including number of leaks and pad test among 30 females with MS when compared to PFMT alone or PFMT and EMG biofeedback ( McClurg *et al*., 2006). A recent systematic review showed that PFMT alone or in conjunction with other interventions among people with MS is also considered to be effective and classified as a first-line treatment approach ( Tornic & Panicker, 2018). Of concern, across numerous studies with non-neurological populations adherence to PFMT has been shown to be the main factor influencing the improvement of urinary symptoms ( Bø *et al*., 2005; Prasad *et al*., 2019; Sacomori *et al*., 2019). One study showed some barriers to adherence among postpartum women included forgetfulness, lack of time, and chores related to childcare ( Sacomori *et al*., 2019). On the other hand, another study showed that using simple reminder methods could improve the adherence rate of PFMT and consequently improve urinary symptoms ( Prasad *et al*., 2019). Further studies are needed to assess adherence to PFMT among people with MS and urinary symptoms and the influence of adherence on urinary symptoms in MS.

The European Association of Urology (EAU), American Urology Association (AUA), and UK National Institute for Care and Clinical Excellence (NICE) recommend combined therapeutic interventions of PFMT and behavioural intervention for non-neurogenic OAB symptoms ( Nambiar & Lucas, 2014) with no clear recommendations for neurogenic bladder symptoms. Intra-detrusor Onabotulinum toxin A (BoNT-A) injection is the most effective therapeutic intervention that classified as a second-line intervention for neurogenic bladder according to EAU guidelines ( Nambiar & Lucas, 2014; Tornic & Panicker, 2018). Despite the reported efficacy of this intervention among neurogenic population and particularly people with MS in improving subjective urinary symptoms and urodynamic parameters ( Cruz *et al*., 2011; Denys *et al*., 2017; Fowler *et al*., 2009; Kalsi *et al*., 2007; McCombe *et al*., 2009; Woodwar, 2004), several adverse events and limitations have been reported including; generalised muscle weakness, the need for repeated injections, increased risk of urinary tract infections (UTIs), increased risk of post-void residual (PVR) due to decline in bladder contractility resulting in the need of clean intermittent catheterization (CIC) afterwards, and rare but severe side effects were also reported such as respiratory problems ( Fowler *et al*., 2009; Groen *et al*., 2016; Kalsi *et al*., 2007; Panicker & Fowler, 2015; Stöhrer *et al*., 2009; Tornic & Panicker, 2018). Although the literature reported the required dosage for BoNT-A injection is 100 unit ( Mehnert *et al*., 2010), the needed dosage for each treatment session is still debatable ( Sahai *et al*., 2010).

Neuromodulation is another available intervention for neurogenic storage symptoms among people with MS. This intervention may include non-invasive, semi-invasive or an invasive surgical procedure. Non-invasive neuromodulation techniques include electrical stimulation of peripheral nerves such as pudendal nerve, dorsal genital nerves, and/ or tibial nerve. Although some studies showed a significant improvement of urinary symptoms using pudendal nerve or dorsal genital nerves among non-neurogenic OAB population, there is a need for further large high quality studies ( Jaqua & Powell, 2017; Janssen *et al*., 2017), with a lack of clear efficacy among neurogenic bladder populations.

Tibial nerve stimulation proximal to medial malleolus using adhesive cutaneous electrodes is another form of non-invasive neuromodulation for bladder storage management called transcutaneous tibial nerve stimulation (TTNS). Percutaneous Tibial Nerve Stimulation (PTNS) is a semi-invasive neuromodulation in which needle electrode is used to stimulate the tibial nerve fibres. Therefore, TTNS could be an alternative intervention among people with MS. Recent reviews have shown that tibial nerve stimulation may be effective in managing storage symptoms in people with non-neurogenic and neurogenic bladder with future high quality studies needed among people with MS ( Aharony *et al*., 2017; Panicker *et al*., 2015; Schneider *et al*., 2015; Tornic & Panicker, 2018). The tibial nerve is a mixed nerve containing sensory and motor fibres of L4-S3, and it originates from the same segments of the spinal cord as the innervation to the bladder, pelvic floor, and rectum. The exact mechanism through which tibial nerve neuromodulation potentially treats bladder storage related symptoms is not yet fully understood ( Arya & Weissbart, 2017; Gaziev *et al*., 2013; Joussain & Denys, 2015; Valles-Antuna *et al*., 2017). However, it is believed that stimulation of the tibial nerve with low-frequency pulses at tolerable intensity can produce inhibition of the spinothalamic neurons within the spinal cord, reduce firing input to the pontine micturition centres (PMC), which in turn inhibits the information facilitated by those nerves to the bladder ( Chancellor & Chartier-Kastler, 2000; Choudhary *et al*., 2016; de Groat, 1997; Devane *et al*., 2015; Zecca *et al*., 2016). Neuromodulation may also have supra-spinal effects which has been investigated in human studies by studying somatosensory evoked potentials before and after Percutaneous tibial nerve stimulation (PTNS) and sacral neuromodulation (SNM). The results showed alterations in somatosensory evoked potential ( Finazzi-Agrò *et al*., 2009; Valles-Antuna *et al*., 2017). This means that stimulation at the tibial nerve, results in modifications of synaptic efficiency through the somatosensory pathway providing information on the function of somatosensory cortical structures at the spino-thalamic level. Although people with MS might have scattered lesions at spinal cord level, some studies showed some promising evidence from tibial nerve stimulation intervention for bladder control in MS. Further studies are needed to determine the correlation between spinal cord lesions and tibial nerve stimulation intervention among people with MS and bladder storage symptoms.

Our review of the literature revealed few studies investigating TTNS among neurogenic bladder ( Amarenco *et al*., 2003; Monteiro *et al*., 2014; Perissinotto *et al*., 2015; Valles-Antuna *et al*., 2017). Specific to people with MS, two experimental studies were identified ( de Seze *et al*., 2011; Seth *et al*., 2018). In the uncontrolled experimental study by de Seze *et al*. (2011); the authors recruited 70 people with MS and OAB. All participants were asked to apply TTNS daily for 20 minutes for 12 weeks. This study found significant improvements in urinary related symptoms of urgency, frequency, and nocturia in 82.6% at the end of week 4, and 83.3% improvement at the end of week 12. Urodynamic studies showed significant improvement by increasing maximum cystometric capacity and reflex volume in about half of the participants (51.2%.).

 A second study was a randomised pilot study assessing the safety, acceptability and pilot efficacy of transcutaneous low-frequency TTNS using self-applicating ambulatory skin-adhering device stimulating the tibial nerve called geko ^TM^ at a frequency of 1 Hz. The study compared two different protocol of TTNS 30 min daily vs. 30 min weekly, for 12 weeks between 24 MS participants with OAB to 24 participants with idiopathic OAB. TTNS was shown to be safe, acceptable and significant improvements in urinary symptoms including OAB were reported for both groups ( Seth *et al*., 2015; Seth *et al*., 2018). The electrical stimulation parameters for both groups were at 27 mA current, with 1 Hz current frequency and pulse width was between 70 and 560 µs and was consecutively increased depending upon the maximum tolerable sensory and best sensory-motor response.

The potential efficacy of TTNS among people with MS with urinary symptoms is based on a small number of studies with a large number of authors identifying the absence of high-quality evidence relating to the electrical stimulation parameters, frequency of treatment per week, and total number of sessions ( Booth *et al*., 2018; Burton *et al*., 2012; Schneider *et al*., 2015; Slovak *et al*., 2015; Tudor *et al*., 2020; Zecca *et al*., 2016).

Randomised controlled trials are required to provide a robust evidence-base for using TTNS for neurogenic bladder storage related symptoms in MS. Therefore, there is a need to assess the feasibility of TTNS in storage symptoms among people with MS using, widely available and affordable device, the transcutaneous electrical nerve stimulation (TENS) unit to proceed with a definitive randomised trial.

### Specific objectives

The overreaching aim of this study is to assess whether transcutaneous tibial nerve stimulation (TTNS) is feasible as a treatment for bladder storage symptoms in people with MS. In particular, the study will explore the feasibility and acceptability of TTNS application in people with MS by addressing the following specific objectives:

*The primary objectives are* to a) assess the recruitment and retention rates, b) investigate the completion rate and appropriateness of outcome measures by participants; c) assess safety by reporting the number of participants with adverse events; d) determine participants’ adherence to the treatment protocol, and e) assess participant’s satisfaction with TTNS application.

*The secondary objectives are to* a) evaluate the preliminary effectiveness of TTNS in managing bladder storage symptoms in MS; and to b) determine the effect of TTNS on quality of life in people with MS.

Addressing these objectives will establish a foundation for future definitive randomised trial to explore the efficacy of TTNS on bladder storage symptoms in MS.

## Methods

The CONSORT extension for pilot and feasibility studies will be followed to standardise the conduct and reporting of the study ( Eldridge *et al*., 2016). Ethical approval was obtained from the Faculty of Education and Health Sciences Research Ethics Committee at the University of Limerick [2020_06_07_EHS]. This study was registered with ClinicalTrials.gov on 27 ^th^ August 2020 ( NCT04528784). The trial registration platform will be updated if any changes are made to the trial.

### Study design

A single-arm experimental study to assess the feasibility of TTNS in MS bladder storage symptoms.

### Setting

The intervention will be accessible to community dwelling people with MS in Ireland and self-administered by participants in their own homes.

### Participants

Inclusion criteria: People with a self-reported diagnosis with any type of MS who are ambulatory; aged ≥18 years old; who have at least one bladder storage related symptom such as urinary frequency, urinary urgency, nocturia, with or without incontinence; and who are willing to give written informed consent.

People with MS will be excluded if they have: indwelling urethral catheter; indwelling suprapubic catheter; urologic disease including bladder malignancy; diabetic mellitus; pregnant women or plan to be pregnant during the study time; recent pelvic related surgery <1 year; pacemaker or other metallic internal devices; urinary tract infections (UTIs) during recruitment phase.

### Participant identification and consent

The recruitment plan is twofold: 1) Open recruitment for people with MS through MS Irelands communication channels for two months duration. This will employ the same procedures as that of our published protocol for a previous qualitative study ( Al Dandan *et al*., 2019). A co-investigator (HD) will send recruitment letters (Appendix A, extended data ( Al-Dandan, 2020)), study information sheets, and screening forms (Appendix B, extended data ( Al-Dandan, 2020)) to a gatekeeper at MS Ireland who will send the emails to all people with MS and who will place the study notifications on their social medial channels. 2) In addition, our recruitment strategy will include a convenience sample of people with MS from an existing cohort of people with MS who have already participated in a qualitative telephone interview study of urinary problems. The protocol for that study, including recruitment strategies, are described in detail elsewhere ( Al Dandan *et al*., 2019).

Interested and eligible participants will be invited to contact a member of the research team including the principal investigator (KR) or co-investigators (SC) or (HD) using the contact methods provided in the email. Once a participant contacts any member in the research team, a call will be arranged via MS Ireland’s Zoom for Healthcare account with (HD) to review eligibility and provide details of the full procedure of the study including the protocol of the nerve stimulation intervention and the outcome measures and give prospective participants the opportunity to ask questions. Participants will be made aware that they have the full right to withdraw at any stage without any explanation. Those who elect to participate will be provided with the link via email to a survey hosted on Qualtrics Survey Software to read and sign the informed consent form, complete demographic data (Appendix C, extended data ( Al-Dandan, 2020)) including the Patient Determined Disease Steps (PDDS) ( Learmonth *et al*., 2013), the International Consultation of Incontinence Questionnaire-Overactive bladder (ICIQ-OAB), King’s Health Questionnaire, the 3- day bladder diary (Appendix D, extended data ( Al-Dandan, 2020)) , adverse events report diary (Appendix E, extended data ( Al-Dandan, 2020)), and adherence to treatment protocol (Appendix F, extended data ( Al-Dandan, 2020)). The 3-day bladder diary, ICIQ-OAB, and King’s Health Questionnaire are to be filled twice during the study; at base line before commencing the intervention (week 0) and post commencing the intervention at the end of (week 6). Diaries including adverse events report diary and adherence to treatment protocol diary are to be filled throughout the study from (week 0) till the end of (week 6) to document any observation related to the application of the intervention. The Questionnaire for Acceptability (Appendix G, extended data ( Al-Dandan, 2020)) is to be filled at the end of the intervention at the end of (week 6) to allow the researcher to determine the participant’s satisfaction level with application of TTNS.

Once participants provide the signed consent form and complete the required outcome measures, a call will be arranged using MS Ireland Zoom Healthcare account to provide details in how to apply the nerve stimulation and allow an opportunity to ask questions. After the call, all participants will be emailed a short video showing the nerve stimulation application as a reminder in how to apply the TENS unit.

The nerve stimulation unit will be posted to them by an MS Ireland’s staff member. In this package, a stamped addressed envelope will also be provided to facilitate return of the nerve stimulation unit.

During the Zoom video call participants will be given an option for paper copy of diaries instead of downloadable documents via Qualtrics Survey Software. In this scenario, HD will inform the interested participants that the hard copies of the diaries will be posted with the TENS unit.

Participants will be called by HD on a weekly basis using MS Ireland Zoom Healthcare account to check adherence to treatment protocol, any reported adverse event, discuss any issues raised up by participants, and at the end of the study will remind them to complete the outcome measures. Responses from participants will be documented to be cross-checked against the self- reported diaries for adherence and adverse events. The study will be discontinued for individual participants if participants report an adverse reaction to the application or use of TTNS.

### TTNS Intervention:

- **Type of equipment**: Stimulation of posterior tibial nerve will be delivered by Transcutaneous Electrical Nerve Stimulation (TENS) unit.
- **Application of TTNS:** based on the anatomical distribution and previous reports: the anode will be positioned between 5–10 cm above medial malleolus and posterior to the edge of the tibia and the cathode will be positioned distally on arch of the foot ( Castel-Lacanal, 2015; Mcguire *et al*., 1983). The accurate location of electrode is considered by contractions of the appropriate muscles innervated by posterior tibial nerve; sensory response by tingling sensation of sole of the foot and / or motor response by flextion of big toe and fanning of the other toes.
- **Patient position**: participant will be advised to lie supine with extended legs or supported sitting with knee extension in order to allow the nerve roots to be free from any compression at knee joint.

Participants will be advised to ensure that their skin is clean skin under the electrode to minimize skin resistance. In cases where participants apply skin mosturiser, they will be advised to wipe the area with alcohol swap or with soap and water and ensure the area is completely dry prior to electrodes application.

- **Duration of Treatment:** A six weeks intervention period has been shown to be effective among a neurogenic population ( Monteiro *et al*., 2014) and preferable among participants who has been interviewed during phase II study *( Al Dandan et al., 2019)*
- **Side of Lower leg**: based on physiology of the central nervous system control of the bladder we would use the left ankle for the application of TTNS because it has been shown that the right side of the brain is the predominant active area in micturition ( de Groat, 1997; Sand & Sand, 2013).
- **Frequency:** 10 HZ. It has been shown that low frequency of 10HZ is effective in inhibiting sympathetic nervous system that might normalize bladder functions ( Robertson *et al*., 2006).
- **Intensity:** the intensity of stimulation will be at the sensory and motor threshold by tingling sensation on sole of the foot with flexion of big toe and /or fanning of other toes. The stimulation should not be painful ( Zecca *et al*., 2014).
- **Pulse duration**: 200 microseconds, it is important to be long enough to allow action potentials to leave the hyperpolarization zone induced by anodic phase ( Vargas Luna *et al*., 2017). It is consistent with previous studies.
- **Stimulus duration:** 30 minutes
- **Frequency of treatment**: 3 times/ week for 6 weeks

### Outcomes

**Primary outcome measures**. A) Feasibility will be assessed by the proportion of participants who: are recruited to the study, complete assigned outcome measures, complete the 6-week intervention, and are lost to follow up. Also, feasibility will be assessed by the overall level of adherence achieved over the study period of 6-weeks through the self-report diary ( Anghel *et al*., 2019). Adverse events will be measured using a self-report diary and will include (but will not be limited to) any device related skin redness at the site of stimulation, discomfort at the stimulation site that might persist post treatment session. B) Acceptability (satisfaction) of TTNS application will be assessed using a self-report questionnaire developed by the researchers containing 5-point Likert scales: 1= “strongly disagree”; 2= “disagree”; 3= “neutral”; 4= “agree”; 5= “strongly agree”.

**Secondary outcome measures** include the followings:

- The storage symptoms will be assessed by ICIQ-OAB ( Abrams *et al*., 2018; Zappavigna & Carr, 2015). It has grade A for validity, reliability and responsiveness to change established with rigour on one data set. The total score ranges from 0 to 16 with higher values indicating increased symptom severity. Bother scales are not incorporated in the overall score ( Abrams *et al*., 2006).
- Severity of frequency, nocturia, and incontinence will be assessed by using a standard 3-day bladder diary ( Jimenez-Cidre *et al*., 2015; Nambiar & Lucas, 2014). Number of episodes of frequency, nocturia, and incontinence/ 72 hours will be calculated and compared from baseline with higher values indicating increased symptom severity.
- Severity and intensity of urgency will be assessed using Patient Perception of Intensity of Urgency Scale (PPIUS) ( Notte *et al*., 2012). Number of urgency episodes and severity of urgency/72 hours will be calculated and compared from baseline. The total score ranges from 0 to 4 with higher values indicating increased symptom severity.
- Quality of Life (QoL) will be evaluated by using King’s Health Questionnaire ( Nambiar & Lucas, 2014; Okamura *et al*., 2009; Pat Ray *et al*., 2003). A change from baseline of 5 points or higher on most of the King’s Health Questionnaire domains represents a clinically meaningful improvement in health-related quality of life after treatment ( Kelleher *et al*., 2004).

### Sample size

This is a pilot study and a formal sample size is not computed. We anticipate that a total of 20 participants will be recruited to the pilot study within two months.

### Data management and confidentiality

The applications used in this study including Zoom Healthcare provided by MS Ireland which uses password protected calls with random meeting ID’s and locked rooms after entry. It is accredited as a tele-health communication system. Also, Qualtrics Survey Software (account held by School of Allied Health, Univeristy of Limerick) are accepted to be used for research as a secured and confidential by the University of Limerick. Data will be handled confidentially and will be stored in accordance with Data Protection Policy at the University of Limerick and in line with the Health Research Regulations 2018 and General Data Protection Regulation (GDPR). All participants will be anonymised by given a unique identifying number and will be entered in a password-protected laptop. Data collected will be accessed only by the team of the study including; HD, KR, SC, RG and DM. All data collected during the study will be entered securely and confidentially in the same laptop by HD. The data will be stored for seven years then all electronic files will be deleted permanently. The full protocol and supplementary Materials are available in an open repository. There are no plans to make the participant dataset open access.

### Statistical methods

SPSS software (Version 26.0. Armonk, NY: IBM Corp) will be used for statistical analysis:

• *The primary outcomes* will be analysed using descriptive statistics (numbers, means, and percentage) with 95% confidence interval (CI). Medians and interquartile ranges will be reported where data are not normally distributed.

• *The secondary outcomes* for the scores of ICIQ-OAB and King’s Health questionnaire at the baseline, and at the end of week 6 will be reported as mean scores with SD and changes in scores between baseline, and week 6 will be examined using T-tests to provide a quantitative indicator of the significance of the changes in the assessment measures following stimulation sessions. The non-parametric equivalent tests will be employed where data are non-parametric.

The incidence of nocturia, incontinence and urinary frequency and urgency will be reported across the two groups and differences will be quantified using a Chi-squared test.

The scale of Patient Perception of Intensity of Urgency Scale (PPIUS) will be assessed using descriptive statistics means and standard deviation (SD).

### Dissemination

The findings of the study will be shared with MS Ireland to be distributed to people with MS and healthcare professionals (HCPs). Also, the study will be submitted for publication to a peer reviewed journal.

### Study status

The study is yet to commence. It is due to commence in September 2020

## Conclusion

To the authors’ knowledge, no feasibility studies have been conducted to evaluate the feasibility of TTNS application for managing storage bladder symptoms in MS. It is anticipated that assessing the feasibility for the application of TTNS among people with MS will contribute to proceed with a definitive randomised trial.

## Data availability

### Underlying data

No underlying data is associated with this article.

### Extended data

Open Science Framework: Transcutaneous tibial nerve stimulation for the treatment of bladder storage symptoms in people with multiple sclerosis: Protocol of a single-arm feasibility study.

https://doi.org/10.17605/OSF.IO/Q2N9E ( Al-Dandan, 2020)

This project contains the following extended data:

- Appendices.docx (recruitment letter, screening form, demographic data sheet, 3- day bladder diary, adverse events report diary, adherence to treatment protocol and the questionnaire for acceptability of treatment)

### Reporting guidelines

Open Science Framework: SPIRIT checklist for ‘Transcutaneous tibial nerve stimulation for the treatment of bladder storage symptoms in people with multiple sclerosis: Protocol of a single-arm feasibility study’ https://doi.org/10.17605/OSF.IO/Q2N9E ( Al-Dandan, 2020)

Data are available under the terms of the Creative Commons Zero “No rights reserved” data waiver (CC0 1.0 Public domain dedication).

## References

[ref-1] Abrams P Andersson KE Apostolidis A: 6th International Consultation on Incontinence. Recommendations of the International Scientific Committee: evaluation and treatment of urinary incontinence, pelvic organ prolapse and faecal incontinence. *Neurourol Urodyn.* 2018;37(7):2271–2272. 10.1002/nau.23551 30106223

[ref-2] Abrams P Avery K Gardener N: The international consultation on incontinence modular questionnaire: www.iciq.net. * J Urol.* 2006;175(3 Pt 1):1063–1066; discussion 1066. 10.1016/S0022-5347(05)00348-4 16469618

[ref-3] Abrams P Cardozo L Wagg A: Incontinence 6th Edition. ICI-ICS. International Continence Society, Bristol,2017 Reference Source

[ref-4] Aharony SM Lam O Corcos J: Evaluation of lower urinary tract symptoms in multiple sclerosis patients: Review of the literature and current guidelines. *Can Urol Assoc J.* 2017;11(1–2):61–64. 10.5489/cuaj.4058 28443147 PMC5403674

[ref-5] Al-Dandan HB: Transcutaneous tibial nerve stimulation for the treatment of bladder storage symptoms in people with multiple sclerosis: Protocol of a single-arm feasibility study.2020 10.17605/OSF.IO/Q2N9E PMC7578569 33117961

[ref-6] Al-Dandan HB Coote S Mcclurg D: Prevalence of Lower Urinary Tract Symptoms in People with Multiple Sclerosis: A Systematic Review and Meta-analysis. *Int J MS Care.* 2020;22(2):91–99. 10.7224/1537-2073.2019-030 32410904 PMC7204365

[ref-7] Al-Dandan HB Galvin R Mcclurg D: Management strategies for lower urinary tract symptoms (LUTS) among people with multiple sclerosis (MS): a qualitative study of the perspectives of people with MS and healthcare professionals [version 1; peer review: 2 approved]. *HRB Open Res.* 2019;2:31. 10.12688/hrbopenres.12960.1 32296749 PMC7140768

[ref-8] Amarenco G Ismael SS Even-Schneider A: Urodynamic effect of acute transcutaneous posterior tibial nerve stimulation in overactive bladder. *J Urol.* 2003;169(6):2210–2215. 10.1097/01.ju.0000067446.17576.bd 12771752

[ref-9] Anghel LA Farcas AM Oprean RN: An overview of the common methods used to measure treatment adherence. *Med Pharm Rep.* 2019;92(2):117–122. 10.15386/mpr-1201 31086837 PMC6510353

[ref-72] Arya NG Weissbart SJ: Central control of micturition in women: Brain-bladder pathways in continence and urgency urinary incontinence. * Clin Anat.* 2017;30(3):373–384. 10.1002/ca.22840 28276096

[ref-10] Bø K Kvarstein B Nygaard I: Lower urinary tract symptoms and pelvic floor muscle exercise adherence after 15 years. * Obstet Gynecol.* 2005;105(5 Pt 1):999–1005. 10.1097/01.AOG.0000157207.95680.6d 15863536

[ref-11] Booth J Connelly L Dickson S: The effectiveness of transcutaneous tibial nerve stimulation (TTNS) for adults with overactive bladder syndrome: A systematic review. *Neurourol Urodyn.* 2018;37(2):528–541. 10.1002/nau.23351 28731583

[ref-12] Browne C Salmon N Kehoe M: Bladder dysfunction and quality of life for people with multiple sclerosis. * Disabil Rehabil.* 2015;37(25):2350–2358. 10.3109/09638288.2015.1027007 25801920

[ref-13] Burton C Sajja A Latthe PM: Effectiveness of percutaneous posterior tibial nerve stimulation for overactive bladder: A systematic review and meta-analysis. * Neurourol Urodyn.* 2012;31(8):1206–1216. 10.1002/nau.22251 22581511

[ref-14] Castel-Lacanal E: Sites of electrical stimulation used in neurology. *Ann Phys Rehabil Med.* 2015;58(4):201–207. 10.1016/j.rehab.2015.05.004 26183200

[ref-15] Chancellor MB Chartier-Kastler EJ: Principles of Sacral Nerve Stimulation (SNS) for the Treatment of Bladder and Urethral Sphincter Dysfunctions. *Neuromodulation.* 2000;3(1):16–26. 10.1046/j.1525-1403.2000.00015.x 22151340

[ref-16] Chapple CR Khullar V Gabriel Z: The Effects of Antimuscarinic Treatments in Overactive Bladder: An Update of a Systematic Review and Meta-Analysis. *Eur Urol.* 2008;54(3):543–562. 10.1016/j.eururo.2008.06.047 18599186

[ref-17] Choudhary M van Mastrigt R van Asselt E: Inhibitory effects of tibial nerve stimulation on bladder neurophysiology in rats. *SpringerPlus.* 2016;5:35. 10.1186/s40064-016-1687-6 26835217 PMC4713404

[ref-18] Cruz F Herschorn S Aliotta P: Efficacy and Safety of OnabotulinumtoxinA in Patients with Urinary Incontinence Due to Neurogenic Detrusor Overactivity: A Randomised, Double-Blind, Placebo-Controlled Trial. * Eur Urol.* 2011;60(4):742–750. 10.1016/j.eururo.2011.07.002 21798658

[ref-19] de Groat WC: A neurologic basis for the overactive bladder. *Urology.* 1997;50(6A Suppl):36–52; discussion 53–6. 10.1016/s0090-4295(97)00587-6 9426749

[ref-20] de Sèze M Raibaut P Gallien P: Transcutaneous posterior tibial nerve stimulation for treatment of the overactive bladder syndrome in multiple sclerosis: results of a multicenter prospective study. *Neurourol Urodyn.* 2011;30(3):306–11. 10.1002/nau.20958 21305588

[ref-21] Denys P Del Popolo G Amarenco G: Efficacy and safety of two administration modes of an intra-detrusor injection of 750 units dysport ^®^ (abobotulinumtoxinA) in patients suffering from refractory neurogenic detrusor overactivity (NDO): A randomised placebo-controlled phase IIa study. *Neurourol Urodyn.* 2017;36(2):457–462. 10.1002/nau.22954 26756554

[ref-22] Devane LA Evers J Jones JFX: A review of sacral nerve stimulation parameters used in the treatment of faecal incontinence. *Surgeon.* 2015;13(3):156–162. 10.1016/j.surge.2014.11.002 25623489

[ref-23] Eldridge SM Chan CL Campbell MJ: CONSORT 2010 statement: extension to randomised pilot and feasibility trials. *Pilot Feasibility Stud.* 2016;2:64. 10.1186/s40814-016-0105-8 27965879 PMC5154046

[ref-24] Finazzi-Agrò E Rocchi C Pachatz C: Percutaneous tibial nerve stimulation produces effects on brain activity: Study on the modifications of the long latency somatosensory evoked potentials. *Neurourol Urodyn.* 2009;28(4):320–324. 10.1002/nau.20651 19090588

[ref-25] Finlayson ML Peterson EW Cho CC: Risk Factors for Falling Among People Aged 45 to 90 Years With Multiple Sclerosis. * Arch Phys Med Rehabil.* 2006;87(9):1274–1279; quiz 1287. 10.1016/j.apmr.2006.06.002 16935067

[ref-26] Fowler CJ Panicker JN Drake M: A UK consensus on the management of the bladder in multiple sclerosis. *J Neurol Neurosurg Psychiatry.* 2009;80(5):470–7. 10.1136/jnnp.2008.159178 19372287

[ref-27] Gajewski JB Schurch B Hamid R: An International Continence Society (ICS) report on the terminology for adult neurogenic lower urinary tract dysfunction (ANLUTD. *Neurourol Urodyn.* 2018;37(3):1152–1161. 10.1002/nau.23397 29149505

[ref-73] Gaziev G Topazio L Lacovelli V: Percutaneous tibial nerve stimulation (PTNS) efficacy in the treatment of lower urinary tract dysfunctions: a systematic review. *BMC Urol.* 2013;13:61. 10.1186/1471-2490-13-61 24274173 PMC4222591

[ref-28] Groen J Pannek J Diaz DC: Summary of European Association of Urology (EAU) Guidelines on Neuro-Urology. *Eur Urol.* 2016;69(2):324–33. 10.1016/j.eururo.2015.07.071 26304502

[ref-29] Haylen BT de Ridder D Freeman RM: An International Urogynecological Association (IUGA)/International Continence Society (ICS) joint report on the terminology for female pelvic floor dysfunction. *Int Urogynecol J.* 2010;21(1):5–26. 10.1007/s00192-009-0976-9 19937315

[ref-30] Janssen DA Martens FMJ De Wall LL: Clinical utility of neurostimulation devices in the treatment of overactive bladder: current perspectives. *Med Devices (Auckl).* 2017;10:109–122. 10.2147/MDER.S115678 28615976 PMC5460621

[ref-31] Jaqua K Powell CR: Where Are We Headed with Neuromodulation for Overactive Bladder? *Curr Urol Rep.* 2017;18(8):59. 10.1007/s11934-017-0711-x 28656519

[ref-32] Jimenez-Cidre MA Lopez-Fando L Esteban-Fuertes M: The 3-day bladder diary is a feasible, reliable and valid tool to evaluate the lower urinary tract symptoms in women. *Neurourol Urodyn.* 2015;34(2):128–32. 10.1002/nau.22530 24264859

[ref-74] Joussain C Denys P: Electrical management of neurogenic lower urinary tract disorders. *Ann Phys Rehabil Med.* 2015;58(4):245–250. 10.1016/j.rehab.2015.07.005 26321622

[ref-33] Kalsi V Gonzales G Popat R: Botulinum injections for the treatment of bladder symptoms of multiple sclerosis. *Ann Neurol.* 2007;62(5):452–7. 10.1002/ana.21209 17890635

[ref-34] Kayes NM Mcpherson KM Schluter P: Exploring the facilitators and barriers to engagement in physical activity for people with multiple sclerosis. * Disabil Rehabil.* 2011;33(12):1043–1053. 10.3109/09638288.2010.520801 20874658

[ref-35] Kelleher CJ Pleil AM Reese PR: How much is enough and who says so? The case of the King's Health Questionnaire and overactive bladder. *BJOG.* 2004;111(6):605–612. 10.1111/j.1471-0528.2004.00129.x 15198790

[ref-36] Learmonth YC Motl RW Sandroff BM: Validation of patient determined disease steps (PDDS) scale scores in persons with multiple sclerosis. *BMC Neurol.* 2013;13:37. 10.1186/1471-2377-13-37 23617555 PMC3651716

[ref-37] Li LF Ka-Kit LG Lui WM: Sacral Nerve Stimulation for Neurogenic Bladder. *World Neurosurg.* 2016;90:236–243. 10.1016/j.wneu.2016.02.108 26960284

[ref-38] Mcclurg D Ashe RG Marshall K: Comparison of pelvic floor muscle training, electromyography biofeedback, and neuromuscular electrical stimulation for bladder dysfunction in people with multiple sclerosis: a randomized pilot study. *Neurourol Urodyn.* 2006;25(4):337–48. 10.1002/nau.20209 16637070

[ref-39] Mccombe PA Gordon TP Jackson MW: Bladder dysfunction in multiple sclerosis. *Expert Rev Neurother.* 2009;9(3):331–40. 10.1586/14737175.9.3.331 19271942

[ref-40] Mcguire EJ Shi-Chun Z Horwinski ER: Treatment of motor and sensory detrusor instability by electrical stimulation. *J Urol.* 1983;129(1):78–79. 10.1016/s0022-5347(17)51928-x 6600794

[ref-41] Mehnert U Birzele J Reuter K: The effect of botulinum toxin type a on overactive bladder symptoms in patients with multiple sclerosis: a pilot study. *J Urol.* 2010;184(3):1011–1016. 10.1016/j.juro.2010.05.035 20643431

[ref-42] Monteiro ÉS De Carvalho LBC Fukujima MM: Electrical Stimulation of the Posterior Tibialis Nerve Improves Symptoms of Poststroke Neurogenic Overactive Bladder in Men: A Randomized Controlled Trial. *Urology.* 2014;84(3):509–514. 10.1016/j.urology.2014.05.031 25168524

[ref-43] Nakipoglu GF Kaya AZ Orhan G: Urinary dysfunction in multiple sclerosis. *J Clin Neurosci.* 2009;16(10):1321–1324. 10.1016/j.jocn.2008.12.012 19560927

[ref-44] Nambiar A Lucas M: Chapter 4: Guidelines for the diagnosis and treatment of overactive bladder (OAB) and neurogenic detrusor overactivity (NDO). *Neurourol Urodyn.* 2014;33 Suppl 3:S21–5. 10.1002/nau.22631 25042139

[ref-45] Nortvedt MW Riise T Myhr KM: Reduced quality of life among multiple sclerosis patients with sexual disturbance and bladder dysfunction. *Mult Scler.* 2001;7(4):231–235. 10.1177/135245850100700404 11548982

[ref-46] Notte SM Marshall TS Lee M: Content validity and test-retest reliability of Patient Perception of Intensity of Urgency Scale (PPIUS) for overactive bladder. *BMC Urol.* 2012;12:26. 10.1186/1471-2490-12-26 22958621 PMC3479079

[ref-47] Okamura K Nojiri Y Osuga Y: Reliability and validity of the Kings Health Questionnaire for lower urinary tract symptoms in both genders. *BJU Int.* 2009;103(12):1673–1678. 10.1111/j.1464-410X.2008.08335.x 19154505

[ref-48] Panicker JN Fowler CJ: Lower urinary tract dysfunction in patients with multiple sclerosis. *Handb Clin Neurol.* 2015;130:371–81. 10.1016/B978-0-444-63247-0.00021-3 26003255

[ref-49] Panicker JN Fowler CJ Kessler TM: Lower urinary tract dysfunction in the neurological patient: clinical assessment and management. *Lancet Neurol.* 2015;14(7):720–732. 10.1016/S1474-4422(15)00070-8 26067125

[ref-50] Pat Ray R Andreas MP Gary JO: Multinational Study of Reliability and Validity of the King's Health Questionnaire in Patients with Overactive Bladder. *Qual Life Res.* 2003;12(4):427–442. 10.1023/a:1023422208910 12797715

[ref-51] Perissinotto MC D'ancona CAL Lucio A: Transcutaneous tibial nerve stimulation in the treatment of lower urinary tract symptoms and its impact on health-related quality of life in patients with Parkinson disease: a randomized controlled trial. *J Wound Ostomy Continence Nurs.* 2015;42(1):94–99. 10.1097/WON.0000000000000078 25549314

[ref-52] Peter TD Peter MM: Neurogenic Bladder. * Adv Urol.* 2012;2012: 816274. 10.1155/2012/816274 22400020 PMC3287034

[ref-53] Prasad TS Subahani J Amirtha B: Assessment of efficacy of home available reminders to aid in the adherence and effectiveness of home-based pelvic floor muscle training in the management of stress urinary incontinence. *IAIM.* 2019;6(9):7–12. Reference Source

[ref-54] Robertson VJ Low J Reed A: Electrotherapy explained: principles and practice. Edinburgh; London: Butterworth-Heinemann Elsevier.2006 Reference Source

[ref-55] Sacomori C Zomkowski K Dos Passos Porto I: Adherence and effectiveness of a single instruction of pelvic floor exercises: a randomized clinical trial. *Int Urogynecol J.* 2019;31(5):951–959. 10.1007/s00192-019-04032-6 31254046

[ref-56] Sahai A Dowson C Khan MS: Repeated Injections of Botulinum Toxin-A for Idiopathic Detrusor Overactivity. *Urology.* 2010;75(3):552–558. 10.1016/j.urology.2009.05.097 20035984

[ref-57] Sand PK Sand RI: The diagnosis and management of lower urinary tract symptoms in multiple sclerosis patients. *Dis Mon.* 2013;59(7):261–268. 10.1016/j.disamonth.2013.03.013 23786660

[ref-58] Schneider MP Gross T Bachmann LM: Tibial Nerve Stimulation for Treating Neurogenic Lower Urinary Tract Dysfunction: A Systematic Review. *Eur Urol.* 2015;68(5):859–67. 10.1016/j.eururo.2015.07.001 26194043

[ref-59] Seth J Haslam C Gonzales G: SINGLE CENTRE RANDOMISED PILOT STUDY OF TWO REGIMENS (30 MINS DAILY OR 30 MINS WEEKLY FOR 12 WEEKS) OF TRANSCUTANEOUS TIBIAL NERVE STIMULATION USING A NOVEL DEVICE FOR TREATING MULTIPLE SCLEROSIS-RELATED OVERACTIVE BLADDER SYMPTOMS. BMJ Publishing Group Ltd.2015 Reference Source

[ref-60] Seth JH Gonzales G Haslam C: Feasibility of using a novel non-invasive ambulatory tibial nerve stimulation device for the home-based treatment of overactive bladder symptoms. *Transl Androl Urol.* 2018;7(6):912–919. 10.21037/tau.2018.09.12 30505727 PMC6256042

[ref-61] Slovak M Chapple CR Barker AT: Non-invasive transcutaneous electrical stimulation in the treatment of overactive bladder. *Asian J Urol.* 2015;2(2):92–101. 10.1016/j.ajur.2015.04.013 29264126 PMC5730708

[ref-62] Stöhrer M Blok B Castro-Diaz D: EAU Guidelines on Neurogenic Lower Urinary Tract Dysfunction. *Eur Urol.* 2009;56(1):81–88. 10.1016/j.eururo.2009.04.028 19403235

[ref-63] Tornic J Panicker J: The Management of Lower Urinary Tract Dysfunction in Multiple Sclerosis. *Curr Neurol Neurosci Rep.* 2018;18(8):54. 10.1007/s11910-018-0857-z 29956001 PMC6022518

[ref-64] Tudor KI Seth JH Liechti MD: Outcomes following percutaneous tibial nerve stimulation (PTNS) treatment for neurogenic and idiopathic overactive bladder. *Clin Auton Res.* 2020;30(1):61–67. 10.1007/s10286-018-0553-8 30074101

[ref-65] Valles-Antuna C Perez-Haro Ml Gonzalez-Ruiz De LC: Transcutaneous stimulation of the posterior tibial nerve for treating refractory urge incontinence of idiopathic and neurogenic origin. *Actas Urol Esp.* 2017;41(7):465–470. 10.1016/j.acuro.2017.01.009 28325529

[ref-66] Vargas Luna Jl Krenn M Mayr W: Optimization of Interphase Intervals to Enhance the Evoked Muscular Responses of Transcutaneous Neuromuscular Electrical Stimulation. *Artif Organs.* 2017;41(12):1145–1152. 10.1111/aor.12921 28567858

[ref-67] Vitkova M Rosenberger J Krokavcova M: Health-related quality of life in multiple sclerosis patients with bladder, bowel and sexual dysfunction. *Disabil Rehabil.* 2014;36(12):987–992. 10.3109/09638288.2013.825332 23962192

[ref-68] Woodwar S: Current management of neurogenic bladder in patients with MS. *Br J Nurs.* 2004;13(7):362–370. 10.12968/bjon.2004.13.7.12679 15150474

[ref-69] Zappavigna C Carr LK: Validated Questionnaires for the Evaluation of Urinary Incontinence—Which, When and Why? *Curr Bladder Dysfunct Rep.* 2015;10:138–142. 10.1007/s11884-015-0290-y

[ref-70] Zecca C Digesu GA Robshaw P: Motor and sensory responses after percutaneous tibial nerve stimulation in multiple sclerosis patients with lower urinary tract symptoms treated in daily practice. *Eur J Neurol.* 2014;21(3):506–11. 10.1111/ene.12339 24387787

[ref-71] Zecca C Panicari L Disanto G: Posterior tibial nerve stimulation in the management of lower urinary tract symptoms in patients with multiple sclerosis. *Int Urogynecol J.* 2016;27(4):521–527. 10.1007/s00192-015-2814-6 26245726

