# Supplementary material for: Transcutaneous tibial nerve stimulation for the treatment of bladder storage symptoms in people with multiple sclerosis: Protocol of a single-arm feasibility study
Source: HRB Open Res. 2020 Sep 16;3:66. [Version 1] doi: 10.12688/hrbopenres.13107.1 (PMC7578569; doi:10.12688/hrbopenres.13107.1)
Supplement: Peer review history

# Peer review: Reviewer response for version 1

**DOI:** 10.21956/hrbopenres.14212.r28019

**Published:** 2020-10-20

**Authors:** Ahmed Al-Badr, Noor Almousa

**Affiliations:** 1 King Fahad Medical City, Riyadh, Saudi Arabia; 2 Urogynecology and Pelvic Reconstructive Surgery Division, Department of Obstetrics and Gynecology, Women's Specialised Hospital, Women Specialized Hospital, King Fahad Medical City, Riyadh, Saudi Arabia

Multiple sclerosis is a progressive disease affecting patients between 20 and 40 years of age. It is characterized by multiple lesions and usually a progressive form of bladder dysfunction especially if the plaques are involving the frontal lobe of the cerebral cortex or the lateral columns of the spinal cord. Bladder dysfunction might be the earliest symptom leading to the diagnosis. Approximately 60% of patients with urinary symptoms show detrusor contractions. Whereas 30% of patients might have underactive or areflexic detrusor.

 The use of percutaneous tibial nerve stimulation (PTNS) was found effective in reducing overactive bladder symptoms among idiopathic and neurogenic candidates. This treatment modality is non-invasive, ambulatory; easy to perform, with minimal adverse effects. PTNS remains a reasonable treatment option for these patients.

 The research project is well structured, the ideas are clear and the writing is concise. You managed successfully to show the importance of your research.

 I have a comment regarding the duration of intervention in your study design. The duration of treatment you mentioned is 3 times per week for 6 weeks although it is well known in some studies that a statistically significant improvement was noted on bladder symptoms after 12 weeks of weekly therapy (Peters *et al., *2010). ^1^

 The other point is; are you ruling out any urinary tract infection during the recruitment phase only? Or at the end of treatment as well while filling up the QoL questionnaires? Or weekly just before each PTNS session?

**Regards,**

**Noor Almousa**

**Ahmed Al-Badr**

Is the study design appropriate for the research question?

Yes

Is the rationale for, and objectives of, the study clearly described?

Yes

Are sufficient details of the methods provided to allow replication by others?

Yes

Are the datasets clearly presented in a useable and accessible format?

Yes

Reviewer Expertise:

Pelvic floor dysfunction, prevalence

We confirm that we have read this submission and believe that we have an appropriate level of expertise to confirm that it is of an acceptable scientific standard.

## References

[1] : Randomized trial of percutaneous tibial nerve stimulation versus Sham efficacy in the treatment of overactive bladder syndrome: results from the SUmiT trial. *J Urol*.2010;183(4) : 10.1016/j.juro.2009.12.036 1438-43 10.1016/j.juro.2009.12.036 20171677

# Peer review: Reviewer response for version 1

**DOI:** 10.21956/hrbopenres.14212.r28051

**Published:** 2020-10-05

**Authors:** David Božidar Vodušek

**Affiliations:** 1 Division of Neurology, University Medical Centre Ljubljana, Ljubljana, Slovenia

Neurogenic lower urinary tract dysfunction is common in MS patients with ensuing "storage" and "voiding" symptoms, resulting in risks for the general health outcome, much bother and poor quality of life.

 Neuromodulation is an available intervention for neurogenic storage symptoms among people with MS, optimally using an non-invasive approach, such as electrical stimulation of the tibial nerve (TTNS). Although there is some clinical and theoretical basis to substantiate such therapy, there is a need for large high quality studies.

 This "....Protocol of a single-arm feasibility study" describes a planned study to assess the feasibility of TTNS in storage symptoms among people with MS using a widely available and affordable device (the transcutaneous electrical nerve stimulation (TENS) unit). Such a study is very helpful before proceeding with a definitive randomised trial.

 This is an excellent, well defined proposal for a study of a clinically very relevant problem. Assessing the feasibility for the application of TTNS among people with MS will contribute to proceed with a definitive randomised trial, which is much needed.

 I have only a comment on correct nomenclature: In "TTNS Intervention" there is the statement "...in order to allow the nerve roots to be free from any compression at knee joint". At the knee joint there are NERVES, and not NERVE ROOTS. (See anatomical texts).

Is the study design appropriate for the research question?

Yes

Is the rationale for, and objectives of, the study clearly described?

Yes

Are sufficient details of the methods provided to allow replication by others?

Yes

Are the datasets clearly presented in a useable and accessible format?

Yes

Reviewer Expertise:

Clinical Neurophysiology, Uroneurology, Peripheral neurology.

I confirm that I have read this submission and believe that I have an appropriate level of expertise to confirm that it is of an acceptable scientific standard.
